# Interfacial Polarization in Thermoplastic Basalt Fiber-Reinforced Composites

**DOI:** 10.3390/polym12071486

**Published:** 2020-07-03

**Authors:** Wojciech Ignaczak, Anne Ladegaard Skov, Miroslawa El Fray

**Affiliations:** 1 Department of Polymer and Biomaterials Science, Faculty of Chemical Technology and Engineering, West Pomeranian University of Technology, Szczecin, Al. Piastów 45, 71-311 Szczecin, Poland, wojciech.ignaczak@zut.edu.pl; 2 Department of Chemical Engineering, Danish Polymer Center, Technical University of Denmark, Søltofts Plads 228A, 2800 Kgs. Lyngby, Denmark, al@kt.dtu.dk

**Keywords:** basalt fiber, interface, MWS polarization, thermoplastic composites, polymer blend

## Abstract

The aim of this work was to study the interfacial behavior of basalt-fiber-reinforced thermoplastic blends of polypropylene and poly(butylene terephthalate) (PP/PBT). We examined the effect of two compatibilizers and two basalt fiber (BF) sizings: commercial (REF) and experimental (EXP). Differential scanning calorimetry was used to assess the influence of BFs on the phase structure of obtained composites. Furthermore, dielectric relaxation spectroscopy was used for the first time to non-destructively study the interfacial adhesion within an entire volume of BF-reinforced composites by assessing the α relaxation, DC conductivity, and Maxwell–Wagner–Sillars (MWS) polarization. The fiber–matrix adhesion was further investigated using the Havriliak–Negami model. Using complex plane analysis, the dielectric strength, which is inversely related to the adhesion, was calculated. The composites reinforced with EXP fibers showed significantly lower values of dielectric strength compared to the REF fibers, indicating better adhesion between the reinforcement and blend matrix. Static bending tests also confirmed improved fiber adhesion with EXP fibers, while also suggesting a synergistic effect between compatibilizer and sizing in enhancing interfacial properties. Thus, we conclude that substantially improved adhesion of PP/PBT BF-reinforced composites is the result of mutual interactions of functional groups of blend matrix, mostly from blend compatibilizer, and fiber surface due to sizing.

## 1. Introduction

Over the last several decades, the modification of existing polymeric systems has arguably become even more important than the synthesis of new polymers. Polymer modification can be achieved in various ways, including copolymerization, blending, and/or by adding organic and inorganic compounds, fillers, or fibrous reinforcement. Each approach leads to the improvement of specific physical properties (i.e., mechanical [1,2], thermal [3,4], electrical [5], and/or dielectric properties [6,7,8]). For highly demanding applications, composite materials—thermoplastic composites in particular—are considered most promising, owing to their high strength-to-weight ratios and excellent fatigue resistance [9]. More importantly, the most common technique of processing thermoplastic composites is injection molding, which enables the production of large quantities of parts with complex shapes within strict dimensional tolerances [10]. Over the last several decades, basalt fibers (BFs) have come into consideration as potential reinforcement of composite materials [11,12,13,14] due to their numerous attractive properties. Thermoplastic BF-reinforced composites are a realistic answer to market demands for materials with lower environmental impact, increased recyclability, ease of processing, and lower cost, while maintaining all of the thermal and mechanical benefits offered by composite materials. However, there are a number of challenges limiting the use of BF reinforcement in thermoplastic composites. The main issue is poor adhesion between the BF and thermoplastic polymers, especially non-polar ones like polyolefins, resulting in ineffective load transfer along the fibers and therefore poor mechanical properties. The interfacial properties of BF-reinforced composites (BFRCs) can be improved using many different approaches, including plasma treatment [15] or chemical activation of the fiber surface [16]. Another method to improve the interfacial properties of BFRCs is the so-called fiber sizing. Sizing is a thin, complex coating applied to the fiber surface during manufacture, partly chemically bonded with the fiber [17,18]. Its purpose is to change the surface wettability and lower the interfacial tension, as well as to provide some additional functional groups that can form intermolecular interactions (i.e., PP–*g*–MA moieties) [19]. The amount of the sizing usually accounts for 0.2 to 0.5 wt.% of the fiber and consists of primary components (mainly silanes), wetting and antistatic agents, lubricants, and binders [20,21]. The development and application of fiber sizing is a common part of fiber manufacture and has already proven effective in enhancing adhesion between glass and carbon fibers, and different thermoset/thermoplastic resins [22,23]. However, for BF reinforcement, there remains a lack of unambiguous reports in the literature that comprehensively explain the role of fiber sizing and the nature of interfacial interactions occurring in BF-reinforced thermoplastics.

Thus, motivated by the increasing demand for high-performance composites, including BF-reinforced thermoplastic composites, we aimed to assess the adhesion of BFs impregnated with different sizings (commercial and experimentally modified by the manufacturer) to thermoplastic PP/PBT blend matrix and to discuss the relationship between fiber–matrix interactions and composite performance. Towards this aim, we used the broadband dielectric spectroscopy (BDS) technique to quantify the interfacial adhesion within the entire volume of the polymer blend matrix. To our knowledge, this is the first time this approach has been used to study BF/thermoplastic blend interactions.

## 2. Materials and Methods 

### 2.1. Materials

**Figure 1:**
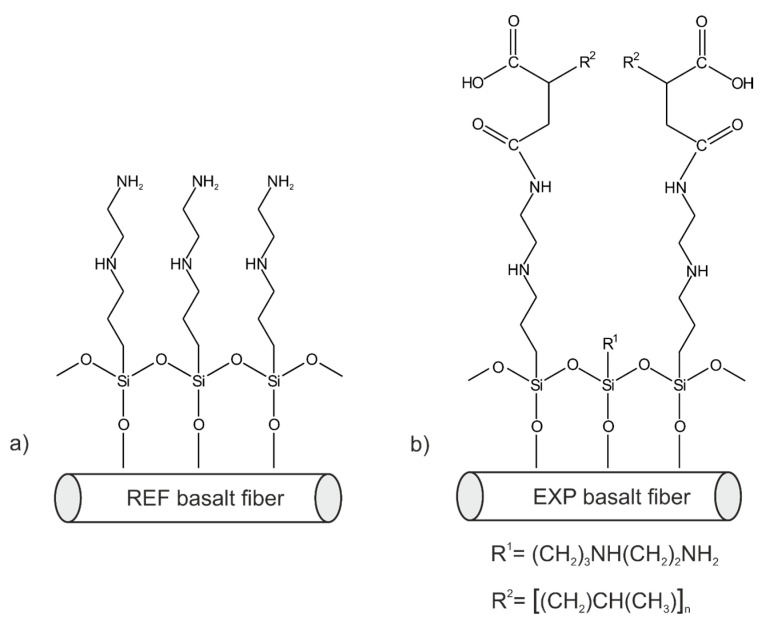
Likely structures of organofunctionalized basalt fiber (BF) surface: (**a**) reference (REF) BFs; and (**b**) experimental (EXP) BFs.

The homopolymer of polypropylene (PP) (Moplen HP 2409) that was used as the PP blend component was purchased from Basell Polyolefins (Płock, Poland). Neat poly(butylene terephthalate) (PBT) (Celanex 1600A) was obtained from Ticona Engineering Polymers (Frankfurt, Germany). The blend components were carefully selected after taking into account their viscosities, as expressed by their mass melt flow indexes (MFIs) at the processing temperature (230 °C) (MFI_PP_:MFI_PBT_, 2.5:3.0 g/10 min). Based on our previous work [24], we used two different compatibilizers to obtain miscible PP/PBT blends of 50/50 wt.% ratio: (1) commercially available SEBS copolymer (Kraton FG 1901GT), obtained from Kraton LLC Polymers (Houston, Texas, USA), with 30 wt.% content of styrene hard segments; (2) PBT–DLA copolymer, consisting of hard segments of butylene terephthalate (30 wt.%) and soft segments of butylene dilinolate (an ester of dimerized fatty acid, here dilinoleic acid, and 1,4-butanediol), synthesized as previously described [25,26]. We added reinforcing materials in the form of two different basalt fiber (BF) types, designated REF and EXP (provided by Isomatex, Gembloux (Isnes), Belgium) [27]. Both BFs used had a diameter of 11 μm and average fiber length of 5 mm, but differed in surface chemistry (the exact coating formula is proprietary): (1) REF was covered with a commercial sizing containing 1° and 2° amine groups, based on 3-(2-aminoethylaminopropyl)trimethoxysilane as coupling agent, which is commonly used in many formulations, especially in thermosetting composites; (2) EXP was covered with an experimental sizing additionally enriched with maleic anhydride moieties (PP–*g*–MA). The graphical representation of the possible arrangements of the molecules on the basalt fiber surface is presented in Figure 1.

### 2.2. Methods

Thermoplastic composites were prepared by a melt-mixing technique [27], using a 20-mm single-screw extruder (*L*/*D* = 30, custom built, West Pomeranian University of Technology, Szczecin, Poland) with a screw speed of 10 rpm, at temperatures ranging from 230 to 240 °C. The chopped, 5 mm BFs were dosed manually because they tended to agglomerate due to their electrostatic properties. The reinforcement content for all of the prepared composites was fixed at 10 wt.%. The polymer composites were then dried in a vacuum oven (air atmosphere, 100 mbar) at 60 °C overnight. For mechanical testing, bar specimens (4 mm × 10 mm) were prepared using a BOY 35A injection molding machine (Dr. Boy GmbH & Co. K, Neustadt-Fernthal, Germany) and conditioned for 48 h at room temperature. 

Differential scanning calorimetry (DSC) analysis was performed with Q100 DSC (TA Instruments Inc., New Castle, Delaware, USA). Experiments were conducted under nitrogen atmosphere (nitrogen 5.0), in the heating–cooling–heating cycle, with samples weighing from 10 to 15 mg. The temperature range was between −50 and 250 °C, with a heating/cooling rate of 10 °C/min. The T_g_ value of each sample was determined from the midpoint of the transition in the second heating run.

Dielectric measurements [28] were performed using a Novocontrol Broadband Dielectric Spectrometer, based on an Alpha analyzer and a Quatro temperature controller BDS 1100 (Novocontrol Technologies GmbH & Co. KG, Montabaur, Germany). The samples were studied in a temperature domain (with 5 °C/min ramp) from −30 to 100 °C, isochronal at 10^1^, 10^2^, 10^3^, 10^4^, and 10^5^ Hz in the case of polymer matrix, and in a frequency domain from 10^−1^ to 10^6^ Hz, isothermally from −30 to 135 °C with 15 °C increment in the case of polymer composites. A circular silver electrode (2 cm in diameter) was sputtered on both surfaces of the sample (1-mm thick) to ensure good electrical contact with the measuring electrodes. The sample was fixed between two external electrodes in the sample holder and placed in a cryostat. A sinusoidal voltage of 1 V was applied, creating an alternating electric field. This resulted in polarization in the sample, oscillating at the same frequency as the electric field, but with a phase angle shift, δ. Measurements of capacitance and conductance were used to calculate the: (1) real part of permittivity (apparent permittivity) ε’, which is proportional to the capacitance and measures the alignment of dipoles; (2) imaginary part of permittivity (loss factor) ε’’, which is proportional to the conductance and represents the energy required to align dipoles and move ions; and (3) dissipation factor, tan δ = ε’’/ ε’. The measured dielectric data were collected and evaluated using WinDETA and WinFIT (Novocontrol Technologies GmbH & Co. KG, Montabaur, Germany) impedance analysis software for non-linear models.

The mechanical properties of the obtained composites were assessed using the 3-point bending test at room temperature with an Instron 3366 (Instron, Norwood, Massachusetts, USA) machine equipped with a 10 kN load cell, according to PN-EN ISO 14125: 2001 standard. For each material, at least 10 specimens were tested. The samples, in the form of bars (4 mm x 10 mm), were tested at a crosshead speed of 10 mm/min (~4 N/s for blends and ~5 N/s for composites). 

## 3. Results and Discussion

### 3.1. Thermal Properties

**Figure 2:**
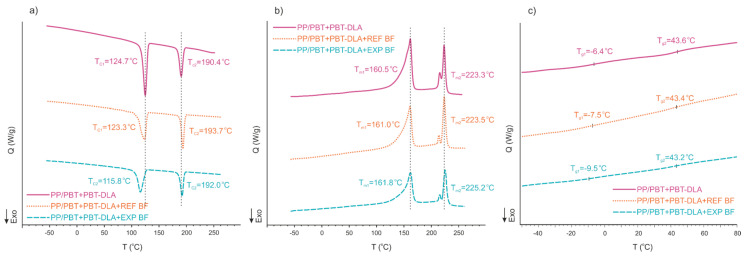
Thermal properties of representative 50/50 PP/PBT composites reinforced with basalt fibers. (**a**) Crystallization; (**b**) Melting; (**c**) Glass transition.

Figure 2 shows representative DSC thermograms (obtained during the cooling and second heating) of 50/50 PP/PBT matrix compatibilized with PBT–DLA and composites reinforced with the two types of BF.

Glass transition temperatures (T_g_), as well as melting points and crystallization temperatures, for all blends and composites studied were determined using Universal Analysis software (TA Instruments Inc., New Castle, Delaware, USA), and are provided in Table 1. 

The addition of BFs influences both the melting and crystallization processes. The addition of 10 wt.% of BF to compatibilized blends resulted in a marked decrease in T_c1_, the crystallization temperature of the PP phase, by 10 °C in the case of EXP fibers, indicating a strong interaction between the PP macromolecules and the surface of BFs enriched, in this case, with PP–*g*–MA moieties. On the other hand, there was a slight increase in T_c2_, the crystallization temperature of the PBT phase of the composites—here BFs serve as nucleating agents. The fiber sizing allows for an even distribution of fibers, thus providing more nucleation sites [10,19,29,30]. Meanwhile, after incorporation of the BF, no significant changes in melting temperatures of the PP phase (T_m1_) were observed, while the melting points of the PBT phase (T_m2_) were shifted towards higher temperatures, likely due to well-developed crystalline structure, stabilized by chopped BFs. 

The minor decrease in the T_g_ of the composites as compared to that of the thermoplastic blend matrix may indicate an increase in the mobility of the polymer chains in the matrix. This decrease is again more pronounced for the PP phase (T_g1_), which may be related to the presence of a low concentration of fibers [31]. Thus, BFs may also act as blend plasticizers.

### 3.2. Dielectric Properties

**Figure 3:**
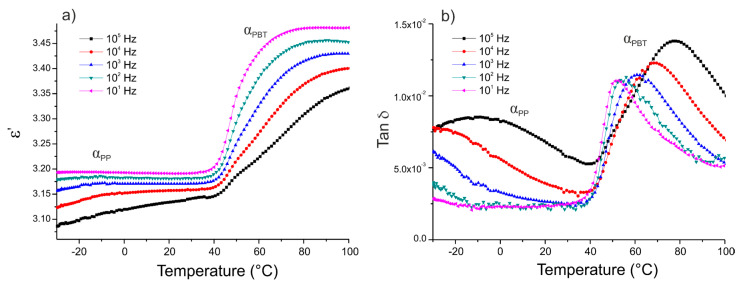
Isochronal runs of: (**a**) real part of the permittivity ε’; (**b**) dissipation factor tan δ as a function of temperature for thermoplastic blend 50/50 PP/PBT.

**Figure 4:**
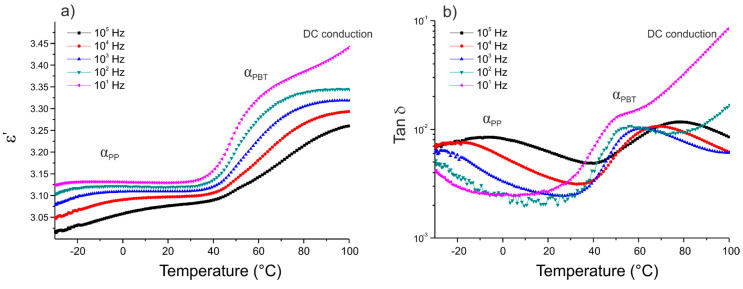
Isochronal runs of: (**a**) real part of the permittivity ε’; (**b**) dissipation factor tan δ as a function of temperature for thermoplastic blend 50/50 PP/PBT+PBT–DLA.

**Figure 5:**
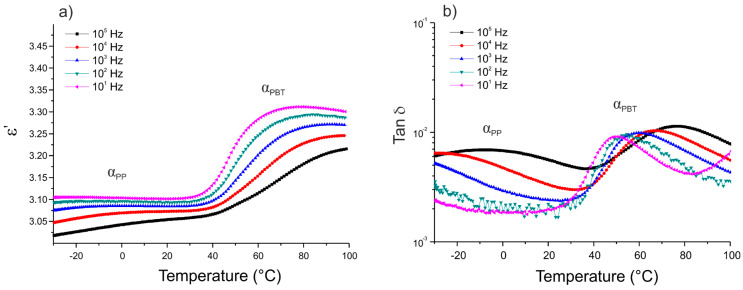
Isochronal runs of: (**a**) real part of the permittivity ε’; (**b**) dissipation factor tan δ as a function of temperature for thermoplastic blend 50/50 PP/PBT+SEBS.

The dielectric characterization of the thermoplastic matrix was conducted isochronally from −30 to 100 °C. Figure 3, Figure 4 and Figure 5 show the variation of the real part of the permittivity ε’ and the dissipation factor tan δ versus temperature for the five frequencies (10^1^, 10^2^, 10^3^, 10^4^, and 10^5^ Hz), for three different 50/50 PP/PBT blends: raw, compatibilized with PBT–DLA copolymer, and compatibilized with SEBS copolymer, respectively. 

The segmental mobility of the polymer chains increased with temperature, leading to an increase in the real part of the permittivity ε’. Each of the thermoplastic matrices exhibited two maximum losses (α relaxation) at low and high temperatures, related to the glass transitions of PP and PBT phase, respectively. The first maximum (α_PP_) was noticeable only at relatively high frequencies (10^5^ and 10^4^ Hz) and can be ascribed to the impurities or methyl side groups and dipoles arising from the oxidation of the PP chain. Among these impurities, the residual catalyst and its potential antioxidant effect may have a more pronounced effect on the dielectric properties of PP [32]. The maximum, marked as α_PBT_, is related to the chain mobility of the polar polyester constituent and occurs in the temperature range of 50 to 80 °C, becoming sharper with increasing frequencies. In the case of PP/PBT blend modified with PBT–DLA copolymer (Figure 4), an additional dielectric relaxation can be observed, marked as DC conduction, most likely due to the presence of some oligomeric and catalyst residues in the synthesized, experimental compatibilizer, which are polarizable at high temperatures. This type of relaxation is also observed in other condensation polyesters derived from either petrochemical or renewable resources [33,34]. The DC conduction effect is much less pronounced in the PP/PBT blend modified with commercial SEBS copolymer (Figure 5), indicating a lower content of low-molecular-weight fraction and catalyst residues, as a result of the employed method of SEBS synthesis (anionic polymerization over organolithium initiators). This method makes it possible to obtain copolymers with low polydispersity indexes and to remove the initiator residues much more effectively using hydrocarbon solvents [35,36]. 

**Figure 6:**
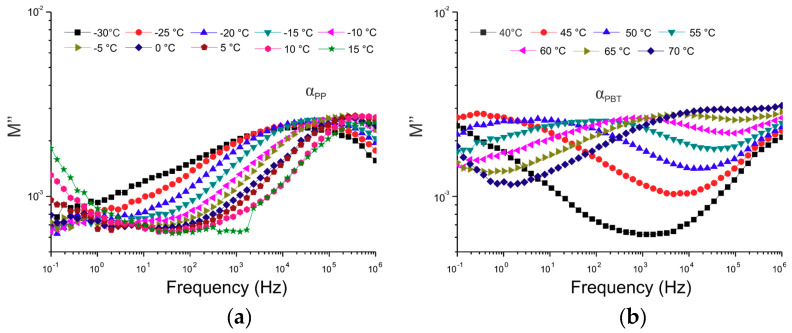
The imaginary part M’’ of the electric modulus versus frequency for representative thermoplastic blend 50/50 PP/PBT+PBT–DLA. (**a**) At lower temperatures (α relaxation of the PP phase); (**b**) At higher temperatures (α relaxation of the PBT phase).

In order to calculate the activation energies as well as relaxation times of α relaxation in the polymer blend matrix, isothermal runs in the frequency domain from 10^−1^ to 10^6^ Hz were conducted over a temperature range from −30 to 100 °C, with an increment of 5 °C. To minimize the effect of DC conductivity, the formalism of the electric modulus [37] was adopted. The electric modulus, M*, is defined by the following equation:

(1) $$M^{*}=\frac{1}{\varepsilon^{*}}=\frac{1}{\varepsilon^{\prime }-i\varepsilon^{\prime \prime }}=\frac{\varepsilon^{\prime }}{\varepsilon^{\prime 2}+\varepsilon^{\prime \prime 2}}+i\frac{\varepsilon^{\prime \prime }}{\varepsilon^{\prime 2}+\varepsilon^{\prime \prime 2}}=M^{\prime }+iM^{\prime \prime }$$

where M′ and M″ are the real and imaginary parts of the electric modulus, respectively. Figure 6 illustrates the isothermal variations of the imaginary part of the electric modulus of the representative thermoplastic matrix.

**Figure 7:**
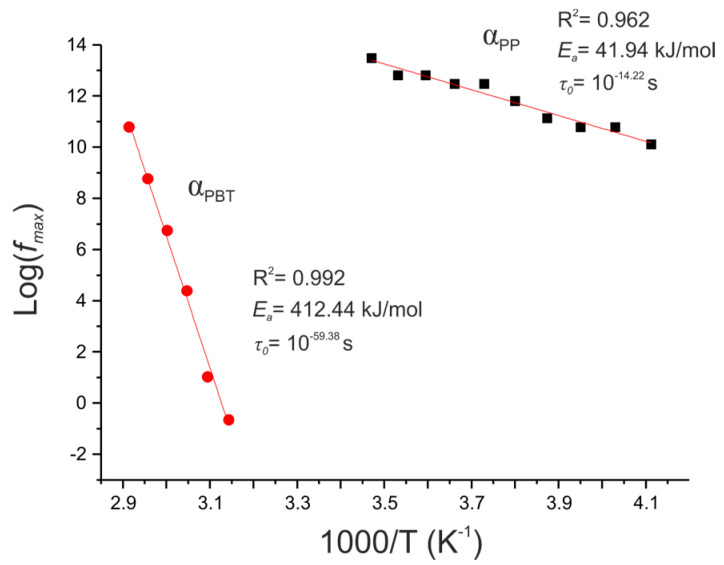
Arrhenius plots of the frequency of M’’ versus the reciprocal temperature for a representative thermoplastic blend 50/50 PP/PBT+PBT–DLA. Lines represent the fitting to Equation (2), representing relaxations for the PP and PBT phases of this blend.

The temperature dependence of the imaginary part of the electric modulus for the representative PP/PBT+PBT–DLA matrix is presented in Figure 7 as an Arrhenius plot. The Arrhenius law was applied to describe the temperature dependence versus maximum frequency (*f_max_*) associated with the maximum of M″ [6,38]. The activation energy of the relaxation processes was calculated according to the equation:

(2) $$f_{max}=f_{0}\exp\left(\frac{-E_{a}}{K_{B}T}\right)$$

where *f*_0_, *E_a_*, *K_B_*, and *T* are the frequency at high temperatures, activation energy of the relaxation process, Boltzmann constant, and temperature, respectively. *E_a_* and *f*_0_ are extracted from the slopes and the intercepts of the plots of log *f_max_* versus reciprocal temperature. The relaxation time *τ*_0_, as $\tau_{0}=\frac{1}{2\pi f_{0}}$, was also calculated. A summary of the calculated activation energies and relaxation times for all of the thermoplastic matrices is given in Table 2.

The values of the activation energies and relaxation times for all of the thermoplastic matrices were comparable, but there was a subtle decrease of the apparent E_a_ and increase in relaxation time for the compatibilized blends. Generally, the reduction of the activation energy is associated with the appearance of some additional interactions between individual components [39] and the induction of chain motions (charge carriers) within a specific volume of polymer matrix, called an interface [40]. This observation is in good agreement with our previous work, where we demonstrated that the miscibility of the PP/PBT blends could be improved by the formation of a specific interface between blend components and two different copolymer compatibilizers [24]. 

**Figure 8:**
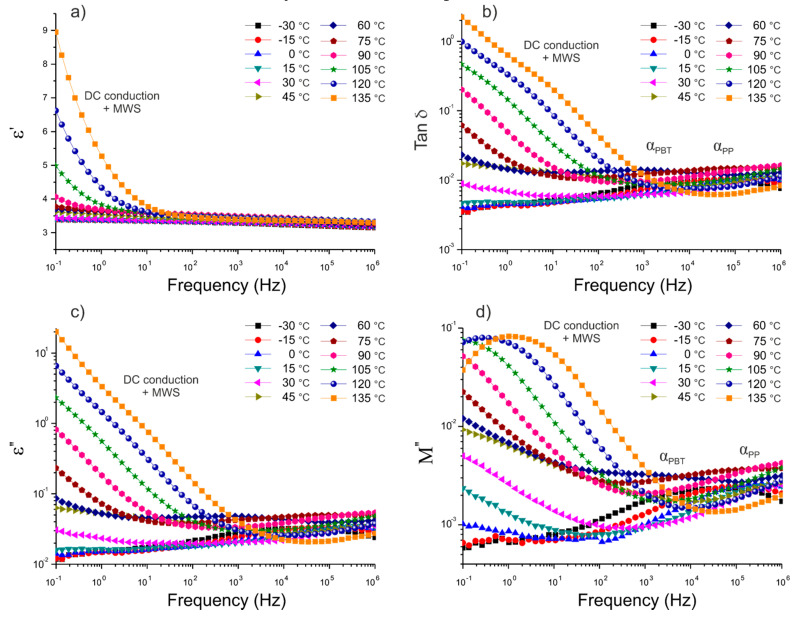
Isothermal runs of: (**a**) real part of permittivity, ε’; (**b**) dissipation factor, tan δ; (**c**) imaginary part of permittivity, ε’’; (**d**) imaginary part of electric modulus, M’’, for PP/PBT+PBT–DLA thermoplastic composite reinforced with REF basalt fibers.

**Figure 9:**
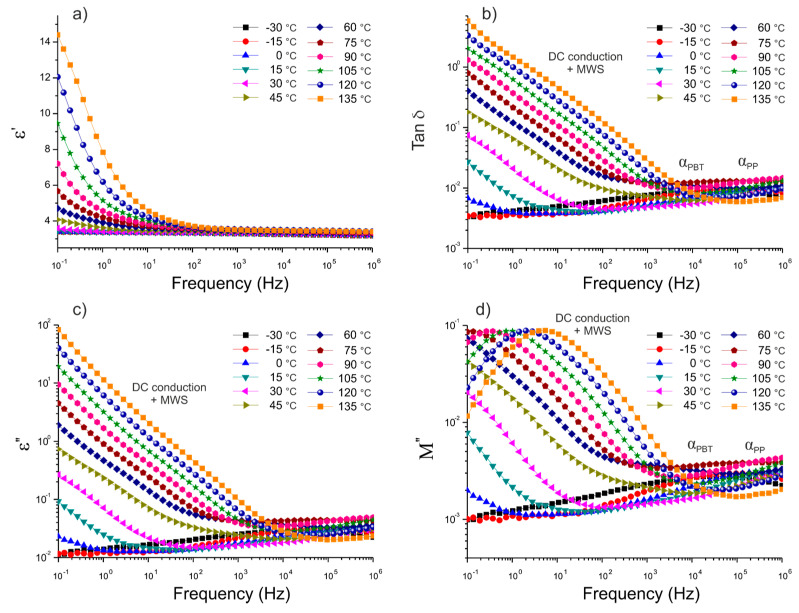
Isothermal runs of: (**a**) real part of permittivity, ε’; (**b**) dissipation factor, tan δ; (**c**) imaginary part of permittivity, ε’’; (**d**) imaginary part of electric modulus, M’’, for PP/PBT+PBT–DLA thermoplastic composite reinforced with EXP basalt fibers.

Next, in order to evaluate the adhesion of BFs to the polymer blend matrix, dielectric characterization of the thermoplastic composites was conducted. Figure 8 and Figure 9 present plots of the frequency dependence of the real (ε’) and the imaginary parts (ε’’) of permittivity, as well as the dissipation factor (tan δ), for the composites reinforced with reference fibers (REF) and experimental ones (EXP), characterized isothermally at different temperatures.

An overall increase in ε’ with temperature at low frequencies was observed for both composites, as a result of an increasing amount of free charges. In the temperature range of 105–135 °C, the juxtaposition of the two effects can be seen, which are related to the DC conductivity and the interfacial polarization process, known as Maxwell–Wagner–Sillars (MWS) polarization [7,40,41]. This phenomenon, comprehensively described in the literature [42,43], is due to the accumulation of free charges at the interface between two or more components differing in electric conductivity. The free charges present in polymeric material as a result of synthesis and thermal processing are anchored in the polymer matrix. Once the temperature is sufficiently high to yield a certain conductivity, the free charges roam due to the applied external electric field. However, when the free charges hit an obstacle in the form of inorganic fibers, with different conductivity, they are polarized at the interface. Furthermore, the ability of these dipoles to relax is associated with the adhesion of the reinforcement to the polymer matrix, and can be numerically evaluated from the dielectric data [44,45]. The complex plane (known as the Argand diagram) representation was used to analyze the nature of this relaxation. It has been well established that the Havriliak–Negami model [46,47] can be used to describe this type of experimental data. Thus, our experimental data were fitted using the Havriliak–Negami equation [46,47] given by the following:

(3) $$\varepsilon^{*}\left(\omega \right)=\varepsilon_{\infty }+\frac{\varepsilon_{s}-\varepsilon_{\infty }}{{\left(1+{\left(i\omega \tau \right)}^{1-\alpha }\right)}^{\beta }}$$

where *α* is the symmetric and *β* is the asymmetric broadening exponent, respectively, ranging from 0 to 1, $\varepsilon_{s}$ and $\varepsilon_{\infty }$ are the dielectric constants for the low- and high-frequency sides of the relaxation, respectively, $\tau =\frac{1}{2\pi f_{max}}$ is the relaxation time, and *ω* is the radial frequency. In the electric modulus formalism, the Havriliak–Negami equation takes the following form:

(4) $$M^{\prime }=M_{s}M_{\infty }\frac{\left[M_{s}A^{\beta }+\left(M_{\infty }-M_{s}\right)cos\beta \varphi \right]A^{\beta }}{M_{s}^{2}A^{2\beta }\left(M_{\infty }-M_{s}\right)M_{s}cos\beta \varphi +{\left(M_{\infty }-M_{s}\right)}^{2}}$$

(5) $$M^{\prime \prime }=M_{s}M_{\infty }\frac{\left[\left(M_{\infty }-M_{s}\right)sin\beta \varphi \right]A^{\beta }}{M_{s}^{2}A^{2\beta }\left(M_{\infty }-M_{s}\right)M_{s}cos\beta \varphi +{\left(M_{\infty }-M_{s}\right)}^{2}}$$

where $M_{s}=\frac{1}{\varepsilon_{s}}$ and $M_{\infty }=\frac{1}{\varepsilon_{\infty }}$

(6) $$A={\left[1+2{\left(\omega \tau \right)}^{1-\alpha }sin\frac{\alpha \pi }{2}+{\left(\omega \tau \right)}^{2\left(1-\alpha \right)}\right]}^{1/2}$$

(7) $$\varphi =arctg\left[\frac{{\left(\omega \tau \right)}^{1-\alpha }cos\frac{\alpha \pi }{2}}{1+{\left(\omega \tau \right)}^{1-\alpha }sin\frac{\alpha \pi }{2}}\right]$$

For each composite, the values of *α*, *β*, $M_{s}$, and $M_{\infty }$ were evaluated using a least squares method, where the following expressions were minimized:

(8) $$\chi_{M^{\prime }}^{2}=\underset{i}{\sum }{\left(M_{th}^{\prime }-M_{exp}^{\prime }\right)}^{2}$$

(9) $$\chi_{M^{\prime \prime }}^{2}=\underset{i}{\sum }{\left(M_{th}^{\prime \prime }-M_{exp}^{\prime \prime }\right)}^{2}$$

**Figure 10:**
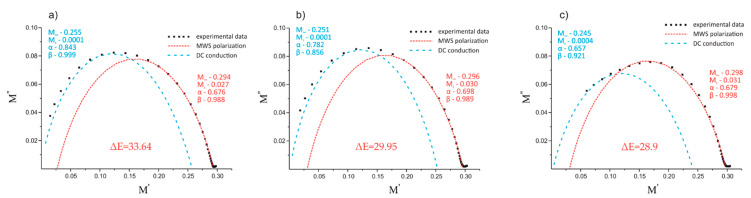
Argand plots of the electric modulus of the composite with REF fibers at 135 °C for 50/50 PP/PBT thermoplastic blends: (**a**) without compatibilizer; (**b**) with PBT–DLA; (**c**) with SEBS.

**Figure 11:**
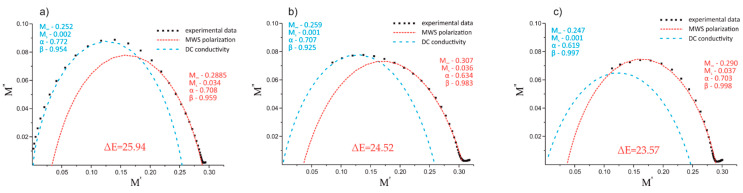
Argand plot of the electric modulus of the composite with EXP fibers at 135 °C for 50/50 PP/PBT thermoplastic blends: (**a**) without compatibilizer; (**b**) with PBT–DLA; (**c**) with SEBS.

Note that the Havriliak–Negami model was not able to account for all the experimental points, most likely due to the nature of both relaxations and DC conduction at lower frequencies [37,48] and their simultaneous superposition. Thus, the best fits of the experimental data with the Havriliak–Negami model were obtained when two overlapping relaxations were modeled: DC conductivity and MWS interfacial polarization [38,39]. As a result, two semicircles were obtained for each composite material in a high temperature range (i.e., 105, 120, and 135 °C). The Argand plots of the thermoplastic composites with 10 wt.% of REF and EXP fibers, based on the three types of 50/50 PP/PBT matrices (uncompatibilized and modified with two different compatibilizers), at maximum temperature (135 °C) are shown in Figure 10 and Figure 11, respectively.

The first semicircle of each figure (blue dashed lines), over approx. 0 < $M^{\prime }$ < 0.25, was assigned to the DC conduction effect, and the second one (red dashed lines), over approx. 0.03 < $M^{\prime }$< 0.3, to Maxwell–Wagner–Sillars polarization in the thermoplastic composites. All of the calculated model parameters for the data collected at 135 °C are provided in Table 3.

The values of α and β indicate that both DC conduction as well as MWS polarization showed notable deviation from the Debye model (α and β equal to 1), most likely due to their mutual superposition. In fact, the deviation from the Debye theory was even greater when additional components were introduced (i.e., additional component on the fiber surface). The change from REF to EXP sizing resulted in a reduction of the α parameter of the DC conduction for all composites: from 0.843 to 0.772 in the case of raw 50/50 PP/PBT blend, from 0.782 to 0.707 for the PBT–DLA modified blend, and from 0.657 to 0.619 for the SEBS modified blend. 

Table 4 shows the interfacial relaxation strength (Δε) of the MWS polarization effect for the thermoplastic composites at the three highest temperatures. Relaxation strength is inversely related to the adhesion between reinforcement and polymer matrix [49], and can be simply calculated from $M_{s}$ and $M_{\infty }$ values ($\Delta \varepsilon =\varepsilon_{s}-\varepsilon_{\infty }$) [50].

For each composite, the dielectric strength Δε of the interfacial polarization increased with increasing temperature. This behavior can be explained by the formation of a greater number of free charges with a tendency to accumulate at the interfaces. This effect is more significant at high temperatures. Furthermore, the increase in temperature results in a greater tendency of dipoles to be polarized, usually accompanied by a subtle shift of the relaxation maxima towards higher frequencies [51]. Overall, the composites reinforced with EXP fibers showed lower values of dielectric strength in comparison with composites reinforced with REF fibers (reduction of Δε from 33.6 to 25.9 at 135 °C for composites with raw 50/50 PP/PBT blend). The reduction in the ability of dipoles to relax, resulting in a lower Δε, can be attributed to better adhesion between the reinforcement and polymer blend matrix. In fact, the greater affinity of EXP BFs to the polymer blend matrix is the result of the presence of maleic anhydride moieties (PP–*g*–MA) on the fiber surface, enhancing the BFs’ adhesion, primarily to the PP phase of the composites. The introduction of compatibilizers (PBT–DLA or SEBS) during the blend preparation step also resulted in a slight decrease in dielectric strength. The presence of a third component having additional functional groups may also lead to improvements in the mutual interaction between the BFs and polymer matrix. Thus, we hypothesize that the functional groups of compatibilizers more willingly interact with hydroxyl/amino groups present on the fiber surface, because the effect of Δε reduction was more pronounced for REF fibers.

These results are in good agreement with our previous work where we used micromechanical testing to examine the effect of EXP BF on the interfacial properties of composites [27]. More importantly, by using dielectric spectroscopy, we were able to assess the fiber–matrix adhesion in the entire volume of the actual composite material, not only in an artificial model system, as was in the case of micromechanical tests.

### 3.3. Mechanical Properties

**Figure 12:**
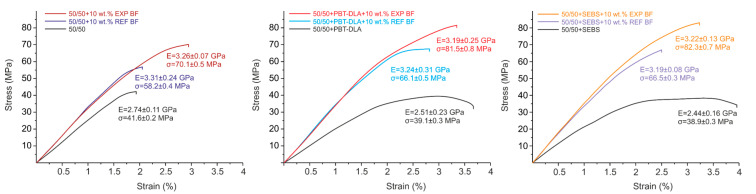
Representative stress–strain curves illustrating the effect of fiber sizing and the matrix (PP/PBT blend containing different compatibilizers) on the mechanical properties of BF-reinforced composites (numerical values represent mean and SD of at least 10 samples).

The results of the three-point bending test of PP/PBT composites are presented in Figure 12. 

The bending stiffness and strength of 50/50 PP/PBT blends decreased after compatibilization, most likely due to the strong influence of the addition of highly elastic compatibilizers with relatively low values of Young’s modulus (several megapascals). Regardless of the matrix, both BF types resulted in a similar increase in flexural stiffness. However, a significant increase in flexural strength (up to 17 MPa) was observed for composites reinforced with EXP fibers when compared to REF fibers—likely due to better stress transfer to the fiber via the polymer matrix. Furthermore, flexural strength values also exhibited an additional impact of applied compatibilizers. Compared to the uncompatibilized PP/PBT blend, both compatibilized PP/PBT blends showed similar, approx. 10% higher and approx. 20% higher, flexural strength when reinforced with REF and EXP fibers, respectively. The addition of both compatibilizing copolymers facilitates additional interactions with the functional polar groups of fiber sizing (e.g., hydrogen bonding or van der Waals interactions), thus improving BF–matrix adhesion. Thus, the trends observed for the mechanical properties of the BF composites reflect the dielectric measurements of interfacial polarization. Furthermore, our results are in good agreement with the literature, where the adhesion of different types of BFs to thermoplastic matrices has been correlated with the polarity of the matrix [19,52].

## 4. Conclusions

The effect of BF sizing on the thermal, dielectric, and mechanical properties of reinforced thermoplastic PP/PBT blend composites was studied. The dielectric measurements show the presence of four relaxation processes in PP/PBT composites, associated with α relaxation of both PP and PBT phases, DC conduction, and MWS polarization. The last phenomenon was studied analytically using the Havriliak–Negami model applying modulus formalism. Based on the dielectric properties, as well as improved mechanical performance, we conclude that the chemical composition of basalt fiber sizing plays a crucial role in forming a strong interfacial fiber–matrix layer. Collectively, our results indicate that the overall adhesion between BFs and PP/PBT matrix is the result of synergistic interactions of functional groups present within the blend matrix, as a result of their compatibilization, and on the fiber surface, due to fiber sizing. Most importantly, our study successfully demonstrates the possibility of assessing the adhesion of BFs to the thermoplastic matrix within the entire volume of material, using the non-destructive dielectric spectroscopy method. Thus, this technique holds promise for the analysis of other BF-reinforced composites.

## Figures and Tables

**Table 1:** Thermal properties of 50/50 PP/PBT composites compatibilized with two different copolymers (PBT–DLA and SEBS) and reinforced with basalt fibers.

Matrix	Fiber	T_c1_ (°C)	T_c2_ (°C)	T_m1_ (°C)	T_m2_ (°C)	T_g1_ (°C)	T_g2_ (°C)
50/50	-	122.9	193.3	160.6	223.3	−6.3	44.0
	REF	120.8	195.8	160.5	223.9	−7.6	42.6
	EXP	120.7	194.5	161.8	225.0	−9.3	43.0
50/50 + PBT–DLA	−	124.7	190.4	160.5	223.3	−6.4	43.6
	REF	123.3	193.7	161.0	223.5	−7.5	43.4
	EXP	115.8	192.0	161.8	225.2	−9.5	43.2
50/50 + SEBS	−	124.5	192.8	160.6	223.3	−6.4	43.4
	REF	123.1	193.9	160.3	224.3	−8.8	43.7
	EXP	113.4	193.6	161.2	225.9	−9.7	44.1

**Table 2:** Activation energies and relaxation times for thermoplastic 50/50 PP/PBT matrix.

PP/PBT Matrix	Relaxation	*E_a_* (kJ/mol)	*τ*_0_ (s)
50/50	α_PP_	51.97	10^−17.83^
	α_PBT_	469.97	10^−63.11^
50/50 + PBT–DLA	α_PP_	41.94	10^−14.22^
	α_PBT_	412.44	10^−59.38^
50/50 + SEBS	α_PP_	43.18	10^−14.51^
	α_PBT_	429.58	10^−61.16^

**Table 3:** Parameters of the Havriliak–Negami model fitted to the experimental data at 135 °C for 50/50 PP/PBT composites reinforced with different BFs.

PP/PBT Matrix	BF	Relaxation	α	β	$M_{s}$	$M_{\infty }$
50/50	REF	conduction	0.843	0.999	0.0001	0.255
		MWS	0.676	0.988	0.027	0.294
	EXP	conduction	0.772	0.954	0.002	0.252
		MWS	0.708	0.959	0.034	0.2885
50/50 + PBT–DLA	REF	conduction	0.782	0.856	0.0001	0.251
		MWS	0.698	0.989	0.030	0.296
	EXP	conduction	0.707	0.925	0.001	0.259
		MWS	0.634	0.983	0.036	0.307
50/50 + SEBS	REF	conduction	0.657	0.921	0.0004	0.245
		MWS	0.679	0.998	0.031	0.298
	EXP	conduction	0.619	0.997	0.001	0.247
		MWS	0.703	0.998	0.037	0.290

**Table 4:** Interfacial relaxation strength Δε of PP/PBT composites reinforced with different BFs.

BF	PP/PBT Matrix	Δε	T (°C)
REF	50/50	7.79	105
	50/50 + PBT–DLA	6.33	
	50/50 + SEBS	6.02	
EXP	50/50	6.83	
	50/50 + PBT–DLA	5.11	
	50/50 + SEBS	5.22	
REF	50/50	22.16	120
	50/50 + PBT–DLA	19.03	
	50/50 + SEBS	18.65	
EXP	50/50	17.44	
	50/50 + PBT–DLA	15.32	
	50/50 + SEBS	15.16	
REF	50/50	33.64	135
	50/50 + PBT–DLA	29.95	
	50/50 + SEBS	28.9	
EXP	50/50	25.94	
	50/50 + PBT–DLA	24.52	
	50/50 + SEBS	23.57	
